# Association between interleukin-6 receptor gene variations and atherosclerotic lipid profiles among young adolescents in Taiwan

**DOI:** 10.1186/1476-511X-10-136

**Published:** 2011-08-12

**Authors:** Nain-Feng Chu, Fu-Hung Lin, Hsien-Chuan Chin, Ye-Jen Hong

**Affiliations:** 1 Department of Community Medicine, Shuang-Ho Hospital, Taipei Medical University, New Taipei City, 235, Taiwan; 2 School of Public Health, National Defense Medical Center, Taipei, 114, Taiwan; 3 Division of Endocrinology, Department of Medicine, Tri-Service General Hospital, National Defense Medical Center, Taipei, 114, Taiwan

**Keywords:** Interleukin-6 receptor gene, Polymorphism, Lipid profiles, Atherosclerotic indexes

## Abstract

**Background:**

To analyze the potential genetic associations between four polymorphisms of interleukin-6 receptor (IL-6R) gene and atherosclerotic lipid profiles among young adolescents in Taiwan.

**Methods:**

Using data from the Taipei Children Heart Study-II - a cross-sectional survey in 2003. After multi-stage sampling, we selected 418 boys and 441 girls with an average age of 13.1 years. We genotyped the subjects for four IL-6R gene polymorphisms (rs4845617 G/A, rs4845623 A/G, rs8192284 A/C, and rs2229238 C/T) using a TaqMan 5' nuclease assay. Lipid profiles, including total cholesterol (CHOL), triglycerides (TG), high density lipoprotein-cholesterol (HDL-C), low density lipoprotein-cholesterol (LDL-C) were measured using standard methods. We also calculated CHOL/HDL-C ratio, LDL-C/HDL-C ratio, and TG/HDL-C ratio as atherosclerotic indexes.

**Results:**

IL-6R rs8192284 A/C and rs2229238 C/T variants showed strong associations with high TG (additive model, OR = 1.58, 95%CI: 1.05-2.37; OR = 1.55, 95%CI: 1.04-2.29, respectively), low HDL-C (additive model, OR = 1.57, 95%CI: 1.03-2.39; OR = 1.68, 95%CI: 1.12-2.52, respectively), and high CHOL/HDL-C (additive model, OR = 1.68, 95%CI: 1.08-2.61, OR = 1.82, 95%CI: 1.18-2.79, respectively) in girls. We inferred five common haplotypes using rs4845617 G/A, rs4845623 A/G, and rs2229238 C/T (GAC, GAT, GGC, AAC, and AAT). In girls, the AAT haplotype was associated with a significant risk of high TG, low HDL-C, high CHOL/HDL-C, and abnormal lipid levels (high TG or low HDL-C) when compared with the GAC haplotype (OR range = 3.08-4.40, all p < 0.05).

**Conclusion:**

The IL-6R rs8192284 A/C and rs2229238 C/T variants are associated with dyslipidemia in girls, but not in boys. The AAT haplotype of the IL-6R gene (rs4845617 G/A, rs4845623 A/G, and rs2229238 C/T) may play an important role in the pathogenesis of dyslipidemia and atherosclerosis in girls.

## Background

Interleukin-6 (IL-6) is a pleiotropic cytokine with important roles in both immunoregulation and non-immune events in a variety of cell types and tissues outside of the immune system [1]. IL-6 binds to the Interleukin-6 receptor (IL-6R), and together they activate the intracellular signaling cascade leading to the inflammatory response [2]. Also, IL-6 has been shown to inhibit lipoprotein lipase activity and stimulate lipolysis, which affects lipid profiles [3], which could contribute to the pathogenesis of atherosclerotic disease [4].

The IL-6R gene is located on human chromosome 1q21 [5], a region reported to be linked to dyslipidemia, metabolic syndrome, and type 2 diabetes [6-8]. In particular, IL-6R rs8192284 A/C (Asp358Ala), in which the polymorphism is localized to a functional domain of the receptor protein [9], was found to correlate with plasma triglyceride levels [6,8].

However, to our knowledge, the relationship between genetic variation in the IL-6R gene and lipid profiles has been rarely investigated in the Asian population, especially among children or young adolescents. The purposes of this study were to examine possible associations between IL-6R polymorphisms (rs4845617 G/A, rs4845623 A/G, rs8192284 A/C, and rs2229238 C/T) and atherosclerotic lipid profiles among young adolescents in Taiwan.

## Methods

### Study design and sampling

The Taipei Children Heart Study-II is an epidemiologic cross-sectional survey aimed at evaluating obesity and cardiovascular-related diseases risk factors among school children in Taipei during 2003. In order to obtain a representative distribution of demographic and lifestyle characteristics, we conducted a cross-sectional survey of junior high school students in Taipei. After a multistage sampling of 85 junior high schools, we randomly selected 1500 school children for this survey. The sampling method and results have been described elsewhere [10]. After considering the study power and excluding missing data, a total of 859 young adolescents (418 boys and 441 girls) with a mean age of 13.1 years (from 12 to 14) were included in the final analyses.

### Data collection

All of the participating young adolescents completed a structured questionnaire detailing their age, gender, puberty development and lifestyle characteristics including cigarette smoking and alcohol consumption. Research technicians measured body weight to an accuracy of 0.1 kg using a standard beam balance scale with subjects barefoot and wearing light indoor clothing. Body height was recorded to the nearest 0.5 cm using a ruler attached to the scale. Waist circumference (WC) was measured to the nearest 0.1 cm at the level of the midpoint between the inferior margin of the last rib and the iliac crest. Hip circumference was measured at its widest point to the nearest 0.1 cm. Segmental Bioelectrical Impedance Analysis was applied to measure body fat percentage to an accuracy of 0.1 percent. The body mass index for each individual was calculated through body weight (kg) divided by the square of their height (m). The Ethical Committee of the Scientific Institute approved this study and informed consent was obtained from the parents and the young adolescents.

We measured serum total cholesterol (CHOL) using the esterase oxidase method [11], triglyceride (TG) using an enzymatic procedure [12], low density lipoprotein-cholesterol (LDL-C) using a multilayer analytical slide method [13], and high density lipoprotein-cholesterol (HDL-C) by an enzymatic method with magnesium precipitation using a Synchron CX5 analyzer (Beckman Instrument, Palo Alto, CA) [14]. We also calculated CHOL/HDL-C ratio, LDL-C/HDL-C ratio, and TG/HDL-C ratio as atherosclerotic indexes. Abnormal lipid profile criteria and atherosclerotic index ratio were determined by age- and gender-specific 90^th ^percentile (10^th ^percentile for HDL-C) cut-off points.

### SNP genotyping

The genotypes of IL-6R rs4845617 G/A, rs4845623 A/G, rs8192284 A/C, and rs2229238 C/T were determined by TaqMan^® ^assay [15]. Assay on demand TaqMan^® ^assays were used for genotyping the SNPs. TaqMan^® ^probes and Universal PCR Master Mix were obtained from Applied Biosystems (Foster City, CA, USA). After amplification, allele specific fluorescence was measured on ABI PRISM^® ^7900 HT Sequence Detector Systems (Applied Biosystems, Foster City, CA, USA).

### Statistical analyses

The distributions of body height, weight, BMI, waist circumference, CHOL, TG, HDL-C, LDL-C, CHOL/HDL-C, LDL-C/HDL-C, and TG/HDL-C with gender specification were described by sample means and standard deviations (SD). The studied children were categorized into subgroups based on their IL-6R genotypes with gender specification.

The differences in lipid profiles across IL-6R genotypes were analyzed by the general linear model (GLM). To determine if IL-6R SNPs are predictors of lipid profiles, a multivariate regression model was applied to assess the association between IL-6R SNPs and these profiles after adjusting for age, cigarette smoking, alcohol drinking and puberty development.

The SNPs were assessed to see if they were in Hardy-Weinberg equilibrium using the ALLELE procedure in SAS/GENETICS release 8.2. We used Haploview software to construct haplotype blocks constituted by "strong LD" markers [16]. The haplotype association analyses were performed by using the HAPLOTYPE procedure in SAS/GENETICS release 8.2. A two-tailed p value less than 0.05 was considered statistically significant.

All statistical analyses were conducted using the statistical package SAS (SAS Institute Inc, Cary, NC, USA).

## Results and Discussion

### Distribution of IL-6R gene polymorphisms

The genotype and allele frequencies of IL-6R gene polymorphisms are shown in Table 1. The frequencies of IL-6R rs4845617 G/A genotypes GG, GA and AA were 29.4, 47.6, and 23.0% for boys and 29.5, 47.8, and 22.7% for girls, respectively. The frequencies of IL-6R rs4845623 A/G genotypes AA, AG and GG were 69.4, 27.7, and 2.9% for boys and 71.2, 26.1, and 2.7% for girls, respectively. The frequencies of IL-6R rs8192284 A/C genotypes AA, AC and CC were 38.5, 48.3, and 13.2% for boys and 33.5, 49.0, and 17.5% for girls, respectively. The frequencies of IL-6R rs2229238 C/T polymorphisms CC, CT and TT were 40.4, 45.9 and 13.7% for boys and 35.4, 45.8 and 18.8% for girls, respectively. There was no significant difference in genotype distribution between boys and girls at these four polymorphisms, and all of them were in Hardy-Weinberg equilibrium. IL-6R rs8192284 A/C and rs2229238 C/T each exhibited (marginal) statistically significant differences in allele frequency with gender (rs8192284 C allele, boys vs. girls = 37.3% vs. 42.0%, p = 0.05; rs2229238 T allele, boys vs. girls = 36.6% vs. 41.7%, p = 0.03, respectively).

**Table 1:** The genotypes and alleles frequencies of IL-6R gene polymorphisms among young adolescents in Taiwan

	Boys (n = 418)		Girls (n = 441)		χ^2 ^test
			
	n	%	n	%	p value
rs4845617 G/A					
Genotype					
GG	123	29.4	130	29.5	0.99
GA	199	47.6	211	47.8	
AA	96	23.0	100	22.7	
Allele					
G	445	53.2	471	53.4	0.94
A	391	46.8	411	46.6	
rs4845623 A/G					
Genotype					
AA	290	69.4	314	71.2	0.84
AG	116	27.7	115	26.1	
GG	12	2.9	12	2.7	
Allele					
A	696	83.2	743	84.2	0.58
G	140	16.8	139	15.8	
rs8192284 A/C					
Genotype					
AA	161	38.5	148	33.5	0.13
AC	202	48.3	216	49.0	
CC	55	13.2	77	17.5	
Allele					
A	524	62.7	512	58.0	0.05
C	312	37.3	370	42.0	
rs2229238 C/T					
Genotype					
CC	169	40.4	156	35.4	0.08
CT	192	45.9	202	45.8	
TT	57	13.7	83	18.8	
Allele					
C	530	63.4	514	58.3	0.03
T	306	36.6	368	41.7	

### Relationship between IL-6R genotypes and lipid profiles

Table 2 shows the relationship between IL-6R genotypes and lipid profiles of young adolescents with gender specification. Those are wild-type carriers of IL-6R SNPs had higher TG levels when compared with boys carrying the minor genotype (for all IL-6R SNPs p = 0.07, marginal significance, with the exception of rs4845623 A/G). In contrast, girls that are minor allele carriers for rs4845623 A/G had higher HDL-C, and girls that are minor allele carriers for rs8192284 A/C and rs2229238 had higher CHOL/HDL-C and LDL-C/HDL-C than those with wild-type genotypes (all p < 0.05).

**Table 2:** The lipid profiles among young adolescents with different IL-6R genotypes

		Wild-type	Heterozygotes	Minor-type	
					
		mean ± s.d.	mean ± s.d.	mean ± s.d.	p^a^
Boys (n = 418)					
rs4845617 G/A		GG (n = 123)	GA (n = 199)	AA (n = 96)	
	CHOL (mg/dl)	158.7 ± 25.9	161.4 ± 28.4	156.7 ± 25.4	0.71
	TG (mg/dl)	70.1 ± 29.9	70.2 ± 34.4	63.5 ± 31.3	0.07
	HDL-C (mg/dl)	49.0 ± 11.0	50.7 ± 11.7	48.8 ± 12.0	0.95
	LDL-C (mg/dl)	80.5 ± 18.2	81.7 ± 19.6	80.6 ± 19.7	0.84
	CHOL/HDL-C	3.38 ± 0.84	3.32 ± 0.85	3.39 ± 1.01	0.85
	LDL-C/HDL-C	1.73 ± 0.57	1.70 ± 0.95	1.77 ± 0.69	0.89
	TG/HDL-C	1.57 ± 0.94	1.52 ± 1.02	1.47 ± 1.08	0.18
rs4845623 A/G		AA (n = 290)	AG (n = 116)	GG (n = 12)	
	CHOL (mg/dl)	160.0 ± 27.2	158.8 ± 26.8	155.3 ± 26.0	0.51
	TG (mg/dl)	68.0 ± 32.0	71.1 ± 33.8	59.3 ± 30.1	0.87
	HDL-C (mg/dl)	49.7 ± 11.6	49.3 ± 11.4	56.7 ± 11.3	0.44
	LDL-C (mg/dl)	81.8 ± 19.2	80.4 ± 19.4	72.0 ± 16.5	0.12
	CHOL/HDL-C	3.36 ± 0.86	3.38 ± 0.96	2.80 ± 0.48	0.24
	LDL-C/HDL-C	1.74 ± 0.59	1.73 ± 0.65	1.31 ± 0.36	0.09
	TG/HDL-C	1.51 ± 0.98	1.61 ± 1.11	1.05 ± 0.46	0.84
rs8192284 A/C		AA (n = 161)	AC (n = 202)	CC (n = 55)	
	CHOL (mg/dl)	158.8 ± 28.6	159.6 ± 26.0	161.3 ± 25.9	0.64
	TG (mg/dl)	69.9 ± 33.5	70.3 ± 33.8	58.7 ± 21.2	0.07
	HDL-C (mg/dl)	48.9 ± 10.6	50.2 ± 12.5	50.6 ± 10.7	0.22
	LDL-C (mg/dl)	80.4 ± 20.2	80.9 ± 18.7	83.9 ± 18.2	0.37
	CHOL/HDL-C	3.38 ± 0.89	3.35 ± 0.93	3.28 ± 0.71	0.46
	LDL-C/HDL-C	1.73 ± 0.60	1.73 ± 0.64	1.72 ± 0.51	0.93
	TG/HDL-C	1.57 ± 1.05	1.56 ± 1.05	1.24 ± 0.62	0.06
rs2229238 C/T		CC (n = 169)	CT (n = 192)	TT (n = 57)	
	CHOL (mg/dl)	158.3 ± 28.9	160.0 ± 25.7	161.2 ± 25.6	0.51
	TG (mg/dl)	70.2 ± 33.3	70.0 ± 34.1	59.5 ± 21.5	0.07
	HDL-C (mg/dl)	49.3 ± 10.9	50.1 ± 12.3	50.1 ± 11.0	0.49
	LDL-C (mg/dl)	79.7 ± 20.7	81.5 ± 18.1	84.0 ± 18.4	0.17
	CHOL/HDL-C	3.35 ± 0.90	3.36 ± 0.91	3.33 ± 0.78	0.96
	LDL-C/HDL-C	1.71 ± 0.61	1.74 ± 0.62	1.75 ± 0.57	0.47
	TG/HDL-C	1.57 ± 1.04	1.56 ± 1.06	1.27 ± 0.63	0.09

Girls (n = 441)					
rs4845617 G/A		GG (n = 130)	GA (n = 211)	AA (n = 100)	
	CHOL (mg/dl)	167.1 ± 26.0	168.7 ± 28.5	169.8 ± 28.2	0.47
	TG (mg/dl)	66.8 ± 27.0	69.6 ± 31.0	74.0 ± 32.1	0.11
	HDL-C (mg/dl)	51.7 ± 11.2	52.5 ± 12.2	49.9 ± 10.3	0.31
	LDL-C (mg/dl)	85.3 ± 18.3	86.7 ± 21.0	87.4 ± 20.6	0.44
	CHOL/HDL-C	3.33 ± 0.70	3.36 ± 0.89	3.52 ± 0.80	0.16
	LDL-C/HDL-C	1.72 ± 0.50	1.74 ± 0.60	1.82 ± 0.55	0.26
	TG/HDL-C	1.37 ± 0.72	1.47 ± 1.01	1.59 ± 0.96	0.09
rs4845623 A/G		AA (n = 314)	AG (n = 115)	GG (n = 12)	
	CHOL (mg/dl)	167.3 ± 27.9	170.5 ± 27.3	178.4 ± 23.7	0.17
	TG (mg/dl)	71.1 ± 31.7	67.5 ± 26.8	58.4 ± 15.3	0.24
	HDL-C (mg/dl)	50.7 ± 11.0	54.2 ± 12.8	51.7 ± 10.0	0.04
	LDL-C (mg/dl)	86.0 ± 20.7	86.8 ± 18.9	93.1 ± 14.2	0.44
	CHOL/HDL-C	3.43 ± 0.85	3.27 ± 0.73	3.54 ± 0.63	0.37
	LDL-C/HDL-C	1.78 ± 0.58	1.68 ± 0.51	1.87 ± 0.46	0.52
	TG/HDL-C	1.53 ± 1.01	1.34 ± 0.66	1.17 ± 0.40	0.07
rs8192284 A/C		AA (n = 148)	AC (n = 216)	CC (n = 77)	
	CHOL (mg/dl)	166.8 ± 26.9	168.6 ± 27.6	171.4 ± 29.3	0.21
	TG (mg/dl)	66.6 ± 24.8	70.5 ± 30.3	73.9 ± 38.0	0.24
	HDL-C (mg/dl)	52.8 ± 11.7	51.6 ± 11.8	49.8 ± 10.2	0.09
	LDL-C (mg/dl)	84.7 ± 18.7	86.7 ± 20.9	89.2 ± 20.3	0.11
	CHOL/HDL-C	3.26 ± 0.66	3.42 ± 0.90	3.56 ± 0.84	0.01
	LDL-C/HDL-C	1.67 ± 0.48	1.77 ± 0.61	1.86 ± 0.54	0.02
	TG/HDL-C	1.34 ± 0.61	1.51 ± 0.99	1.62 ± 1.18	0.10
rs2229238 C/T		CC (n = 156)	CT (n = 202)	TT (n = 83)	
	CHOL (mg/dl)	166.7 ± 27.2	168.6 ± 27.0	171.5 ± 30.2	0.17
	TG (mg/dl)	66.3 ± 24.6	70.9 ± 30.9	73.6 ± 36.9	0.16
	HDL-C (mg/dl)	52.9 ± 11.9	51.5 ± 11.7	49.8 ± 10.4	0.07
	LDL-C (mg/dl)	84.5 ± 18.9	86.7 ± 20.8	89.2 ± 20.5	0.08
	CHOL/HDL-C	3.25 ± 0.66	3.43 ± 0.90	3.56 ± 0.84	0.008
	LDL-C/HDL-C	1.66 ± 0.48	1.78 ± 0.61	1.86 ± 0.54	0.01
	TG/HDL-C	1.33 ± 0.61	1.52 ± 1.01	1.61 ± 1.15	0.06

Abbreviation: CHOL, Cholesterol; TG, Triglycerides; HDL-C, High density lipoprotein-cholesterol; LDL-C, Low density lipoprotein-cholesterol.

^a ^Using GLM test after adjusting for age, cigarette smoking, alcohol drinking, and puberty status.

### IL-6R variants and the risk of abnormal lipid profiles

The logistic regression analyses of IL-6R genotypes on the risk of abnormal lipid profiles and atherosclerotic indexes in young adolescents after adjusting for age, cigarette smoking, alcohol drinking, and puberty status are shown in Table 3. We found significant association of rs4845617 G/A, rs8192284 A/C, and rs2229238 C/T variants with high TG in girls (additive model, OR = 1.59, 95%CI: 1.06-2.37; OR = 1.58, 95%CI: 1.05-2.37; OR = 1.55, 95%CI: 1.04-2.29, respectively). In addition, the minor alleles of IL-6R rs8192284 A/C and rs2229238 C/T were associated with low HDL-C (additive model, OR = 1.57, 95%CI: 1.03-2.39; OR = 1.68, 95%CI: 1.12-2.52, respectively) and high CHOL/HDL-C (additive model, OR = 1.68, 95%CI: 1.08-2.61; OR = 1.82, 95%CI: 1.18-2.79, respectively). The minor allele carriers of rs8192284 A/C and rs2229238 C/T had a greater risk of high TG (OR = 2.21, 95%CI: 1.10-4.44; OR = 2.14, 95%CI: 1.09-4.20, respectively), low HDL-C (OR = 1.98, 95%CI: 0.99-4.00; OR = 2.16, 95%CI: 1.07-4.35, respectively), and high CHOL/HDL-C (OR = 3.17, 95%CI: 1.37-7.30; OR = 3.47, 95%CI: 1.51-8.00, respectively) than wild-type girls in recessive model analyses. However, lipid status in boys was not significantly associated with IL-6R polymorphisms.

**Table 3:** Logistic regression analyses of different IL-6R genotypes on lipid status among young adolescents ^a^

	Model type	rs4845617 G/A		rs4845623 A/G		rs8192284 A/C		rs2229238 C/T	
					
		OR	95%CI	OR	95%CI	OR	95%CI	OR	95%CI
Boys									
High CHOL	additive	0.91	0.55-1.50	1.10	0.58-2.06	0.90	0.53-1.52	0.81	0.48-1.36
	dominant	0.74	0.29-1.86	0.77	0.09-6.39	1.36	0.53-3.50	1.32	0.51-3.38
	recessive	1.01	0.46-2.19	1.18	0.56-2.47	0.70	0.35-1.42	0.58	0.29-1.17
High TG	additive	0.92	0.61-1.40	1.45	0.85-2.46	0.85	0.54-1.34	0.81	0.51-1.27
	dominant	0.95	0.46-1.97	1.56	0.32-7.50	0.39	0.12-1.32	0.38	0.11-1.26
	recessive	0.86	0.45-1.63	1.55	0.83-2.91	1.04	0.56-1.94	0.95	0.52-1.75
High HDL-C	additive	1.02	0.70-1.49	0.98	0.58-1.64	0.78	0.51-1.18	0.84	0.56-1.27
	dominant	1.35	0.72-2.52	0.51	0.06-4.04	0.68	0.28-1.68	0.79	0.34-1.85
	recessive	0.81	0.45-1.45	1.04	0.58-1.89	0.74	0.43-1.28	0.81	0.46-1.40
High LDL-C	additive	0.78	0.49-1.26	0.81	0.42-1.57	0.83	0.50-1.39	0.81	0.49-1.34
	dominant	0.94	0.41-2.15	-	-	1.22	0.48-3.09	1.18	0.46-2.97
	recessive	0.60	0.30-1.21	0.90	0.43-1.90	0.65	0.33-1.27	0.62	0.32-1.22
High CHOL/HDL-C	additive	1.24	0.79-1.93	0.74	0.38-1.43	0.70	0.43-1.16	0.78	0.48-1.27
	dominant	1.39	0.67-2.89	-	-	0.30	0.07-1.26	0.45	0.13-1.51
	recessive	1.28	0.61-2.66	0.81	0.39-1.67	0.79	0.41-1.51	0.86	0.45-1.64
Girls									
High CHOL	additive	1.23	0.79-1.93	0.96	0.51-1.79	1.13	0.71-1.80	1.20	0.77-1.88
	dominant	1.15	0.55-2.43	1.78	0.36-8.79	1.51	0.70-3.27	1.60	0.76-3.38
	recessive	1.55	0.71-3.36	0.85	0.41-1.78	0.97	0.49-1.92	1.07	0.54-2.12
High TG	additive	1.59	1.06-2.37	0.57	0.30-1.09	1.58	1.05-2.37	1.55	1.04-2.29
	dominant	1.77	0.95-3.28	-	-	1.53	0.77-3.02	1.54	0.79-2.99
	recessive	2.00	0.97-4.11	0.59	0.29-1.19	2.21	1.10-4.44	2.14	1.09-4.20
High HDL-C	additive	1.24	0.83-1.86	0.76	0.41-1.41	1.57	1.03-2.39	1.68	1.12-2.52
	dominant	1.24	0.64-2.41	0.73	0.09-5.94	1.67	0.84-3.32	1.90	0.98-3.67
	recessive	1.46	0.74-2.92	0.74	0.37-1.46	1.98	0.99-4.00	2.16	1.07-4.35
High LDL-C	additive	1.08	0.70-1.65	1.01	0.56-1.82	1.10	0.71-1.71	1.22	0.80-1.86
	dominant	0.87	0.41-1.84	0.75	0.09-6.08	0.77	0.33-1.82	0.99	0.45-2.16
	recessive	1.38	0.68-2.83	1.05	0.53-2.05	1.48	0.74-2.96	1.63	0.81-3.25
High CHOL/HDL-C	additive	1.27	0.83-1.94	0.70	0.37-1.36	1.68	1.08-2.61	1.82	1.18-2.79
	dominant	1.38	0.69-2.75	1.75	0.36-8.56	1.35	0.64-2.86	1.61	0.79-3.27
	recessive	1.38	0.68-2.82	0.57	0.27-1.22	3.17	1.37-7.30	3.47	1.51-8.00

Abbreviation: CHOL, Cholesterol; TG, Triglycerides; HDL-C, High density lipoprotein-cholesterol; LDL-C, Low density lipoprotein-cholesterol.

additive model: major-homozygotes v.s. heterozygotes v.s. minor-homozygotes, reference group: major-homozygotes.

dominant model: major-homozygotes/heterozygotes v.s. minor-homozygotes, reference group: major-homozygotes/heterozygotes.

recessive model: major-homozygotes v.s. heterozygotes/minor-homozygotes; reference group: major-homozygotes.

^a ^All abnormal variables defined as age- and gender-specific 90^th ^percentile cut-off point of study variables and OR was adjusted for age, cigarette smoking, alcohol drinking, and puberty status.

### Haplotype analyses of IL-6R gene polymorphisms on the risk of abnormal lipid profiles

**Figure 1:**
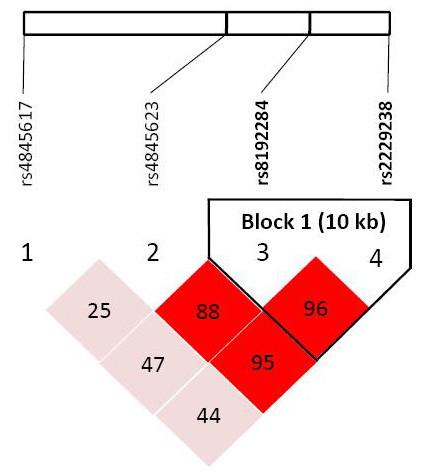
**Haploview linkage disequilibrium (D') display the haplotype structures of IL-6R genes**. Darker shading of cells indicates stronger LD between single nucleotide polymorphisms.

Table 4 shows the haplotype frequencies of the IL-6R gene and their association with abnormal lipid profiles and atherosclerotic indexes. We inferred the haplotypes from the polymorphisms within the linkage disequilibrium block (including IL-6R rs4845617 G/A, rs4845623 A/G, rs8192284 A/C, and rs2229238 C/T). Because rs8192284 A/C and rs2229238 C/T were almost in linkage disequilibrium (r^2^>0.9), only rs2229238 C/T was kept in the haplotype inference, together with the other two SNPs (rs4845617 G/A, rs4845623 A/G) in this linkage disequilibrium block (Figure 1.). Five common haplotypes (frequency >5%) were identified (H1: GAC, H2: GAT, H3: GGC, H4: AAC, H5: AAT) with the three SNPs (rs4845617 G/A - rs4845623 A/G - rs2229238 C/T). Haplotype H5 had a significant risk of high TG, low HDL-C, high CHOL/HDL-C, and abnormal lipid levels (high TG or low HDL-C) when compared with the haplotype H1 in girls (OR = 3.08, 95%CI: 1.07-8.87; OR = 3.30, 95%CI: 1.11-9.85; OR = 4.40, 95%CI: 1.35-14.32; and OR = 3.22, 95%CI: 1.34-7.76, respectively). However, there was still no significant association of abnormal lipid profiles with IL-6R haplotypes in boys.

**Table 4:** Haplotype frequencies of IL-6R gene and associated with lipid status among young adolescents ^a^

	Haplotype^b^	Abnormal	Normal	OR	95%CI	p	Global p
							
		HF	HF				
**Boys**							
High TG	H1	0.35	0.33	1.00	-	-	0.75
	H2	0.08	0.10	0.49	0.08-2.84	0.43	
	H3	0.13	0.10	1.27	0.27-5.91	0.76	
	H4	0.13	0.14	0.68	0.15-2.98	0.61	
	H5	0.24	0.27	0.61	0.20-1.90	0.39	
Low HDL-C	H1	0.39	0.32	1.00	-	-	0.29
	H2	0.07	0.10	0.31	0.06-1.60	0.16	
	H3	0.07	0.11	0.23	0.04-1.18	0.08	
	H4	0.13	0.14	0.47	0.13-1.78	0.27	
	H5	0.24	0.27	0.51	0.18-1.38	0.18	
Abnormal lipid^c^	H1	0.36	0.32	1.00	-	-	0.84
	H2	0.09	0.10	0.64	0.17-2.41	0.51	
	H3	0.09	0.11	0.55	0.14-2.10	0.38	
	H4	0.14	0.14	0.68	0.21-2.18	0.51	
	H5	0.24	0.27	0.64	0.26-1.57	0.33	
High CHOL/HDL-C	H1	0.37	0.33	1.00	-	-	0.19
	H2	0.06	0.10	0.27	0.03-2.29	0.23	
	H3	0.05	0.11	0.16	0.02-1.45	0.10	
	H4	0.19	0.14	1.26	0.32-5.02	0.74	
	H5	0.25	0.27	0.63	0.19-2.05	0.44	
**Girls**							
High TG	H1	0.21	0.30	1.00	-	-	0.04
	H2	0.16	0.13	2.75	0.62-12.26	0.19	
	H3	0.06	0.12	0.47	0.06-3.64	0.47	
	H4	0.17	0.13	3.22	0.68-15.36	0.14	
	H5	0.36	0.28	3.08	1.07-8.87	0.04	
Low HDL-C	H1	0.22	0.30	1.00	-	-	0.13
	H2	0.17	0.12	3.56	0.81-15.53	0.09	
	H3	0.09	0.11	1.35	0.23-8.04	0.74	
	H4	0.12	0.14	1.45	0.24-8.81	0.69	
	H5	0.37	0.28	3.30	1.11-9.85	0.03	
Abnormal lipid	H1	0.22	0.31	1.00	-	-	0.03
	H2	0.16	0.12	2.93	0.85-10.03	0.09	
	H3	0.08	0.12	0.91	0.20-4.07	0.90	
	H4	0.15	0.13	2.57	0.67-9.85	0.17	
	H5	0.36	0.27	3.22	1.34-7.76	0.009	
High CHOL/HDL-C	H1	0.21	0.30	1.00	-	-	0.08
	H2	0.17	0.12	4.66	0.97-22.38	0.05	
	H3	0.10	0.11	2.05	0.32-13.28	0.45	
	H4	0.11	0.14	1.97	0.29-13.46	0.49	
	H5	0.38	0.28	4.40	1.35-14.32	0.01	

Abbreviation: CHOL, Cholesterol; TG, Triglycerides; HDL-C, High density lipoprotein-cholesterol; LDL-C, Low density lipoprotein-cholesterol; HF, Haplotype frequency

^a ^All abnormal variables defined as age- and gender-specific 90^th ^percentile cut-off point of study variables

^b ^Five common haplotype frequencies of IL-6R rs4845617 G/A, rs4845623 A/G, and rs2229238 C/T (H1: GAC, H2: GAT, H3: GGC, H5: AAC, H6: AAT) at least 5% and H1 as reference group.

^c ^Abnormal lipid includes high TG or low HDL-C

## Discussion

In this study, the minor allele of IL-6R rs8192284 A/C and IL-6R rs2229238 C/T variants were associated with a higher risk of abnormal lipid profiles in girls. The girls with the AAT haplotype of the IL-6R gene (rs4845617 G/A, rs4845623 A/G, and rs2229238 C/T) had significant risk of high TG, low HDL-C, high CHOL/HDL-C, and abnormal lipid levels (high TG or low HDL-C). However, there was almost no statistical association between IL-6R variants and lipid profiles in boys.

IL-6 binds to its receptor (IL-6R), and together they activate the intracellular signaling cascade leading to the inflammatory response [17]. Previous studies have reported that IL-6 is associated with lipid metabolism [3,18]. It is involved in production of TG, decreases lipoprotein lipase activity and monomeric lipoprotein lipase in plasma, which contributes to increased macrophage uptake of lipids. In atheromatous plaques and fatty streaks, smooth muscle cells and macrophage foam cells express IL-6, may be an important role for this cytokine in the progression of atherosclerosis [4]. Some studies have indicated that elevated TG may correlate with IL-6R gene polymorphisms [6,8]. Our data reveals that there is a borderline association of the major allele (the A allele) of IL-6R rs8192284 A/C and increased TG levels in boys, which is similar to other findings [6,8]. In contrast, the minor alleles of the IL-6R SNPs were related to high dyslipidemia risk in girls. The relationship between IL-6R rs8192284 A/C variants and high TG risk was inconsistent with previous findings [6,8]. In Spain, a study of middle-aged Caucasians indicated that carriers of the A allele of the IL-6R rs8192284 A/C polymorphism had higher plasma TG levels than those with the CC genotype [6]. In the Guangzhou Biobank Cohort Study-Cardiovascular Disease (GBCS-CVD) sub-cohort study, the A allele of IL-6R rs8192284 A/C also was associated with increased TG levels among aged Chinese [8].

The possible reasons for inconsistent results in girls are as follows. First, there is strong evidence that IL-6 levels increase with age [19], and some studies have evaluated alterations in the frequency of IL-6 SNPs with age [20,21]. Moreover, IL-6 and insulin action have had conflicting results in different tissues (organs), such as adipose tissue, skeletal muscle, and liver [22]. IL-6 and lipid metabolism might potentially contribute to different adipose or skeletal muscle distribution in children and adults. Our study was focused on children, so the subjects were younger than in the Spanish and GBCS-CVD studies (mean ages 43.3 and 59.4 years, respectively). We suggest that differences in the frequencies of genotypes or alleles may, in part, be responsible for the discrepancies in the results of these studies, but that age differences between the populations should also be taken into consideration. Secondly, previous studies have shown that there are gender differences in IL-6 production [23], therefore IL-6 or IL-6R polymorphisms modifying IL-6 levels might potentially contribute to sex disparity. In IL-6 polymorphisms, females with the CC genotype of IL-6 -174 G/C have an earlier onset of type 1 diabetes when compared with IL-6 -174G allele females and all males [24]. Of studies focused on IL-6R polymorphisms, some have analyzed females (in relation to IL-6 levels, CRP levels, bone mineral density, obesity, hyperandrogenism, preterm birth, type 2 diabetes, and melanoma) [25-32], and another concentrated on males (studying the relationship between IL-6R polymorphisms and obesity) [33]. Most analyses of IL-6R SNPs have not investigated (or at least have not reported) gender differences. We found that there was no gender difference in IL-6R rs8192284 A/C genotype frequency distribution, but that there was a borderline significant disparity in allele frequency distribution, which is similar to recent studies [33,34]. In addition, previous studies have found that estrogen modulation and the inherently greater amount of adipose tissue in females relative to males may be responsible for the observed differences in IL-6 production [35]. Our data show that a relationship between IL-6R SNPs and lipid status is almost only observed in girls, for both genotype and haplotype analyses. The IL-6R genotype or allele distribution of different genders may be responsible for these results. The gender-specific role of IL-6R SNPs in the pathology of dyslipidemia should be further investigated.

Haplotypes are more likely than single polymorphisms to identify disease associations because they reflect complete gene structure. IL-6R rs8192284 A/C is a well-known functional polymorphism in exon 9 of the IL-6R gene which results in an amino acid substitution (Asp358Ala) [36]. After calculations using Haploview software, we determined that rs8192284 A/C and rs2229238 C/T were almost in LD (LD = 0.97, r^2 ^= 0.91). The LD was similar to that calculated for rs2228145 G/T (corresponding to Asp358Ala substitution) and rs2229238 C/T in a Pima Indian study (LD = 0.98, r^2 ^= 0.78) [37]. Using Haploview, we picked rs4845617 G/A, rs4845623 A/G, and rs2229238 C/T for haplotype analysis. We found that carriers of the AAT haplotype had a significantly greater risk of dyslipidemia than carriers of the major allele haplotype (GAC). The results indicate that haplotype analysis can indicate a higher dyslipidemia risk than single polymorphism analysis. The haplotype effects within the IL-6R SNPs might explain the discrepancies between our results and previous studies, which examined SNPs separately. Further studies might explore how IL-6R haplotype influences lipid profiles.

Several limitations of our study should be noted. First, we did not control the potential confounders of food intake and physical activity. These factors correlate with plasma lipids and IL-6 levels [38], so that may affect IL-6R gene expression and disturb the regulation of IL-6 and lipid metabolism. In an adult population, a similar study of IL-6R SNPs and lipid profiles may yield biased or confounded results if subjects are on a special diet, exercise, or are being treated for lipid-related disorders, which may also alter lipid levels. However, children of these age groups are rarely on special diets, exercise, or treatment regimens for dyslipidemia. Secondly, the measurement errors in assessing the biological variables of lipid profiles are likely to be minimal. Any error is likely to be random and only attenuate our results. Thirdly, the sample size limits the statistical power of this study. We may not confirm absolutely which SNP is the real functional variant, nor rule out the possibility of minor variants within our SNPs. Moreover, we did not detect serum levels of IL-6 to investigate the relationship between the IL-6R SNPs and the serum level of IL-6. We could not observe a direct association between the IL-6 levels and lipid profiles for IL-6R variants. In the future, may be worth investigating possible associations between IL-6R SNPs and serum levels of IL-6 or sIL-6R in children.

The notable findings of our study were that the IL-6R rs4845617 G/A, rs4845623 A/G, and 2229238 C/T polymorphic markers were chosen to construct the haplotype, which differed from that used in previous studies, and that they showed a significant association with lipid status. This is also the first study to investigate the relationship between IL-6R SNPs and lipid profiles in young adolescents. Many biological processes and different tissues contribute to circulating IL-6 levels. If the IL-6R gene polymorphisms relate to IL-6 and lipid metabolism, they could be used as stable biological markers to detect lipid-related disorders and be very helpful for the prevention of these diseases.

## Conclusions

In summary, IL-6R rs8192284 A/C and rs2229238 C/T variants associated with dyslipidemia were presented among young adolescents, especially in girls, in Taiwan. The AAT haplotype of IL-6R gene SNPs rs4845617 G/A, rs4845623 A/G, and rs2229238 C/T may play an important role in the pathogenesis of dyslipidemia and atherosclerosis in girls. Further mechanistic studies are warranted to explore how IL-6R SNPs exert their effect on lipid metabolism. This information may suggest ways of tailoring strategies for the prevention of dyslipidemia and atherosclerosis according to the genetic make-up of each individual patient.

## Competing interests

The authors declare that they have no competing interests.

## Authors' contributions

NFC carried out the design, coordination and conduction of the study, participated in the data collection and analysis, participated in drafted the manuscript. FHL performed the data collection, statistical analyses, and participated in drafted the manuscript. HCC participated in the data collection, SNP analysis and statistical analysis. YJH carried out the design and coordination of the study. All authors read and approved the final manuscript.
